# Synthetic antimicrobial peptides Bac-5, BMAP-28, and Syn-1 can inhibit bovine respiratory disease pathogens *in vitro*

**DOI:** 10.3389/fvets.2024.1430919

**Published:** 2024-08-12

**Authors:** Santiago Cornejo, Cassandra Barber, Merrilee Thoresen, Mark Lawrence, Keun Seok Seo, Amelia Woolums

**Affiliations:** ^1^Department of Pathobiology and Population Medicine, College of Veterinary Medicine, Mississippi State University, Mississippi State, MS, United States; ^2^Department of Comparative Biomedical Sciences, College of Veterinary Medicine, Mississippi State University, Mississippi State, MS, United States

**Keywords:** BRD, AMP, AMR, cattle, Bac-5, BMAP-28, Syn-1, cathelicidins

## Abstract

Mass treatment with antibiotics at arrival has been the mainstay for bovine respiratory disease (BRD) control but there is an increase in antimicrobial-resistant bacteria being shed from treated cattle. BRD is a disease complex that results from the interaction of viruses or bacteria and susceptible animals with inappropriate immunity. With bacteria being the only feasibly treatable agent and the emergence of antimicrobial resistance, decreased efficacy of commonly used antibiotics could threaten livestock health. There is a need for new antimicrobial alternatives that could be used to control disease. Naturally occurring antimicrobial peptides (AMP) have been proposed to address this need. Here we tested the effect of bovine myeloid antimicrobial peptide-28 (BMAP-28), a synthetic BMAP-28 analog Syn-1, and bactenecin 5 (Bac-5) on *Mannheimia haemolytica* (Mh) using a quantitative culture method and the broth microdilution method to determine minimum inhibitory and bactericidal concentrations (MIC and MBC). We also tested the antiviral effect of these AMP against bovine herpes-1 (BHV-1) and bovine respiratory syncytial virus (BRSV) using the Reed and Muench method to calculate the viral titers after treatment. We demonstrated that BMAP-28 and Syn-1 can inhibit Mh growth and BMAP-28 can inhibit replication of BHV-1 and BRSV. Moreover, we showed that BMAP-28 and Bac-5 can be used together to inhibit Mh growth. When used alone, the MIC of BMAP-28 and Bac-5 was 64 and 128 μg/mL respectively, but when applied together, their MIC ranged from 0.25–16 for BMAP-28 and 8–64 μg/mL for Bac-5, resulting in a decrease in concentration of up to 256 and 16-fold, respectively. The synergistic interaction between those peptides resulted in concentrations that could be well tolerated by cells. Our results demonstrate that bovine cathelicidins could be used as alternatives to antimicrobials against BRD pathogens. These findings introduce a path to discovering new antimicrobials and determining how these peptides could be tailored to improve cattle health.

## Introduction

Bovine respiratory disease (BRD) is considered the costliest disease in beef cattle in North America, with an annual estimated cost of approximately $1 billion (1). This disease complex results from the combination of bacterial colonization in the upper and lower respiratory tract of susceptible animals with inadequate immunity, increased stress, and concomitant infection with viral agents (1–3). BRD losses are due not only to the death of affected cattle, loss of performance, and increased labor expense but also to the required treatments (4–6). As bacteria are the only practically treatable component of this disease complex, there has been a historical reliance on the use of antibiotics to treat or prevent BRD (7–9). Bacteria usually recovered from cattle with BRD are categorized as Gram-negative, with *Mannheimia haemolytica* (Mh) being one of the predominant isolates (10). Viral agents usually associated with disease, among others, are bovine respiratory syncytial virus (BRSV) and bovine herpes virus 1 (BHV-1), which can cause disease by themselves or in combination with bacterial pathogens (11). Although antimicrobial usage has been the mainstay of BRD treatment, research in recent years has shown an increase in antimicrobial resistance (AMR) in bacterial agents of BRD (7, 12–15). This increasing evidence of AMR, and the emergence of multidrug-resistant pathogens, threatens animal health and welfare (9, 16).

Antimicrobial peptides (AMP) are a large class of naturally occurring antimicrobials with important implications in the innate immune defense of different organisms (17, 18). Within this class, the cathelicidin family can be found, which is present in mammalian, bird, insect, amphibian, and fish species (19, 20). Their wide antimicrobial spectrum includes Gram-negative and positive bacteria, fungi, parasites, and various viruses, including those involved in respiratory infections (21). Within the bovine species, 10 cathelicidin genes have been discovered with 7 of them coding for functional proteins: bactenecin 1 (Bac-1), Bac-5, Bac-7, indolicidin, bovine myeloid antimicrobial peptide-27 (BMAP-27), BMAP-28, and BMAP-34 (22, 23). Despite the evidence that cathelicidins can exert antimicrobial effects that could be clinically useful, their efficacy against BRD agents has not been yet reported.

While antimicrobials can induce the development of resistance in microbes, the relatively nonspecific electrostatic interaction established between cationic AMP and the negatively charged microbial membrane reduces opportunities for the development of resistance, thus making them good candidates for the development of new treatments, with no AMR promotion (24).

Although AMP have useful characteristics, there are some risks related to their use to treat or prevent disease. It has been shown that AMP induce a degree of cytotoxicity in mammalian cells (25). To address this issue, researchers have developed synthetic alternatives using naturally occurring AMP as a template, resulting in peptides with enhanced activity and decreased cytotoxicity toward host cells. One example is the AMP Syn-1, synthesized by Sahoo et al., which was designed using the bovine cathelicidin 5 (BMAP-28) as a template (26).

The objective of this study was to test the antimicrobial effects of synthetic bovine AMP against *Mannheimia haemolytica* (Mh), BHV-1, and BRSV. For this purpose, we chose to evaluate cathelicidin 2 (Bac-5) and cathelicidin 5 (BMAP-28) to represent the naturally occurring AMP, and Syn-1, a modified synthetic version of BMAP-28.

## Materials and methods

### Synthetic peptides

The amino acid sequences of all synthesized peptides are shown in Table 1. BMAP-28 and Bac-5 were purchased from a commercial manufacturer (FabGennix International Inc., Frisco, TX), reconstituted in phosphate buffered saline (PBS) without calcium and magnesium chloride according to the manufacturer’s recommendations, and aliquots were stored at −80^°^C until use. Syn-1 was purchased from a different manufacturer (GenScript, Piscataway, NJ), reconstituted in water according to the manufacturer’s recommendations, and aliquots were stored at −80^°^C until use.

**Table 1:** Peptides used in the study.

Peptide	Sequence	Structure	MW	Net charge
BMAP-28 (Cath 5)	GGLRSLGRKILRAWK KYGPI IVPIIRI-NH2	α-helical	3,075	+7
Bac-5 (Cath 2)	RFRPPIRRPPIRPPFYPPFRPPIRPPIFPPIRPPFRPPLGPFP-NH2	PPII	5,148	+9
Syn-1	GG**F**RS**F**GRKIFRAWKKYG-NH2	α-helical	2,161	+6

MW, molecular weight; BMAP-28, bovine myeloid antimicrobial peptide 28; Cath 5, cathelicidin 5; Bac-5, bactenecin 5; Cath 2, cathelicidin 2; PPII, Type II poly-L-proline conformation.

### Bacteria and viruses used

*Mannheimia haemolytica* field isolates 28-32R53, 25-75R5, and 35-248 were used in this research. All isolates were isolated from recently weaned beef calves with BRD signs from a stocker operation in Mississippi and identified using a Sensititre ARIS HiQ AST System (ThermoFisher Scientific cat. no. V4000, Waltham, MA). Mh 28-32R53 and 25-75R5 were obtained in 2016 and Mh 35-248 in 2019. Strain 35-248 was multidrug-resistant as defined by resistance to 3 or more antimicrobial classes. Sampling cattle to collect these isolates was approved by the Mississippi State University Institutional Animal Care and Use Committee (IACUC), protocols #16-692 and #18-529. All isolates were propagated in Mueller Hinton broth (BD Difco™ cat. no. BD 275730 distributed by Fisher Scientific, Hampton, NH), stored at −80°C in 50% glycerol, and passage numbers 2–4 were used for the experiments. Bovine herpes virus-1 Cooper (Colorado-1, VR-864) strain and bovine respiratory syncytial virus (375, VR-1339) strain were obtained from the American Type Culture Collection (ATCC) and propagated in bovine kidney cells as previously described (48, 49).

### Testing effects of AMP on bacteria

#### Antimicrobial effect of AMPs

Mh 35-248 was grown in Mueller Hinton broth and quantified on Mueller Hinton agar (BD Difco™ cat. no. BD 225250, distributed by Fisher Scientific, Hampton, NH) by quantitative culture. Two Mh inoculums, one at 10^3^ and the other at 10^5^ cfu/mL were treated with a final concentration of BMAP-28, Syn-1, or Bac-5 at either 10 or 100 μg/mL. Mh inoculum used in each treatment was quantified before treatment was added (Pre Treatment) and at 0, 12, and 24 h post-treatment. Mh grown in medium was used as negative antimicrobial control and bacteria treated with a final concentration of 100 μg/mL of florfenicol (Nuflor™ distributed by ValleyVet, Marysville, KS) was used as a positive antimicrobial control. Mh inoculum concentrations were achieved by serial dilutions of a 0.5 McFarland standard prepared using a Sensititre Nephelometer (ThermoFisher Scientific cat. no. V3011, Waltham, MA).

#### Antimicrobial susceptibility testing

Mh isolates 28-32R53, 25-75R5, and 35-248 grown in Mueller Hinton broth were tested against AMP using a modified protocol to determine the minimum inhibitory concentration (MIC) adapted to cationic antimicrobial peptides. All assays were performed in polypropylene 96 well plates and dilutions were prepared in polypropylene 1.5 mL tubes.

Bacterial suspensions at 0.5 McFarland standard were prepared for each Mh strain and diluted to reach the desired inoculum concentration of 1 × 10^6^ cfu/ml. Fifty μL of bacterial inoculum were tested against 50 μL of either AMP or florfenicol so that the final antimicrobial concentrations ranged from 0.25–256 μg/mL. Plates were incubated at 35^+/−^2°C, 5% CO_2_ for 18–24 h. MICs were defined by direct observation of the wells as the lowest concentration of antimicrobial agent that completely inhibited organism growth (27).

Three 10 μL aliquots from the lowest antimicrobial well with visual growth and three 10 μL aliquots from each of the next three wells with no visual growth were transferred to separate Mueller Hinton agar plates to confirm the presence or absence of bacterial growth. Plates were read after 24 h incubation at 35^+/−^2^o^C, 5% CO_2,_ and the minimum bactericidal concentration (MBC) was defined as the lowest concentration of antimicrobial where no bacterial growth was visually seen (27).

#### Calculation of fractional inhibitory concentration index

The fractional inhibitory concentration index (FICi) of the AMP in combination with florfenicol were calculated using the following formula “FICi = FICa + FICb,” where FICa corresponds to the MIC of the AMP divided by the MIC of the combination of AMP and florfenicol, and FICb corresponds to the MIC of the florfenicol divided by the MIC of the combination of AMP and florfenicol (28). With the known MIC for each AMP and florfenicol, a checkerboard assay was designed with serial dilutions of the AMP and florfenicol, starting from 2 concentrations above the MIC for each antimicrobial. Plates were incubated at 35^+/−^2^o^C, 5% CO2 for 18–24 h and MICs for the combination of AMP and florfenicol were defined as stated before.

#### Cell growth

Unless otherwise indicated, bovine kidney cells (BK) were used for viral and cytopathic assays and were grown in Dulbecco’s Modified Eagle Medium (DMEM) with 4.5 g/L glucose, L-glutamine, and sodium pyruvate (CORNING cat. no. 10-012-CM, Corning, NY) supplemented with 10% FBS (Avantor® Seradigm Ultimate Grade Fetal Bovine Serum distributed by VWR, cat. no. 97068-101, Radnor, PA) and 1 mM Gibco™ Glutamax™ (cat. no. 35050061, Invitrogen, Waltham, MA).

### Testing effects of AMP on virus

#### Viral cytopathic effect

Using the 4x magnification of an inverted microscope, BHV-1 and BRSV cytopathic effect (CPE) were identified by observation of typical cytolysis and syncytia formation, respectively.

#### Viral TCID50 assay

BK cells seeded at 10^4^ cells per well of a 96 well plate were infected with the untreated virus at 10^3^ or 10^4^ TCID_50_ units, or virus pre-incubated for 2 h at 37^o^C with a final concentration of 100 or 10 μg/mL of each AMP. After 5 days post-infection and 7 days post-infection for BHV-1 and BRSV, respectively, TCID_50_ units were calculated using the Reed and Muench method (29).

#### Cytopathic effects of peptides on cells

The MTT [3-(4,5-dimethylthiazol-2-yl)2,5-diphenyltetrazolium bromide] assay kit (Biotium Inc. cat. no. 30006, Freemont, CA), following the manufacturer’s instructions, was used to test the cytotoxicity of the AMP on bovine cells. Briefly, a 96-well tissue culture plate seeded with 10^4^ BK cells per well in 90 μL of media was incubated for 24 h at 37^o^C with 10 μL of each treatment. Treatments include AMP at 100 or 10 μg/mL per well, and florfenicol (Nuflor™) at 100 or 3 μg/mL per well. Negative control cells were treated with 1x PBS (BMAP-28 and Bac5 solvent), water (Syn-1 solvent), and media, respectively. For a positive cytotoxicity control, a 5% dimethyl sulfoxide (DMSO) solution was used. After the 24 h incubation, 10 μL of a ready-to-use MTT solution was added to each well of treated BK cells and incubated at 37°C for 4 h. Two hundred μl of DMSO (≥99.7%, Sigma-Aldrich cat. no. 34869, St. Louis, MO) were then added to each well to reach a final volume of 310 μL, mixed by pipetting, and the absorbance was measured at 570 nm using a plate reader. The background was read at 630 nm and subtracted from signal absorbance to obtain normalized values.

### Statistical analysis

For data analysis, SAS 9.4 (SAS Institute INC., Cary, NC) was used, all data were log-transformed, and normality was assessed by residual analysis with a *p*-value of less than 0.05 considered significant. The statistical inferences, for the bacterial quantitative culture, were made by comparison of least square means with a linear mixed model for each AMP against each Mh concentration and trial as a random effect, two trials were performed. For viral TCID_50_, the Reed and Muench method was used to calculate the TCID_50_ units. For normally distributed data, statistical inferences were made by comparison of least square means with a linear mixed model for each AMP against each viral strain and concentration using the trial as a random effect, two trials were performed. For non-normally distributed data, intra-cluster correlation (ICC) was calculated by dividing the between cluster variance (variance of mean trial 1 and mean of trial 2) by the within variance (variance of all data + the variance between clusters), if value equals to 0, trial data were considered one cluster and statistical inferences were made by the Kruskal-Wallis test with a multiple comparison analysis (2 clusters). For the cytopathic effect of the AMP in mammalian cells, statistical inferences were made between treatments and supplemented media control by comparison of the least square mean with a linear mixed model and trial as a random effect, two trials were performed.

## Results

### Bacterial assays

#### Effect of BMAP-28, Bac-5, and Syn-1 on *Mannheimia haemolytica*

**Figure 1:**
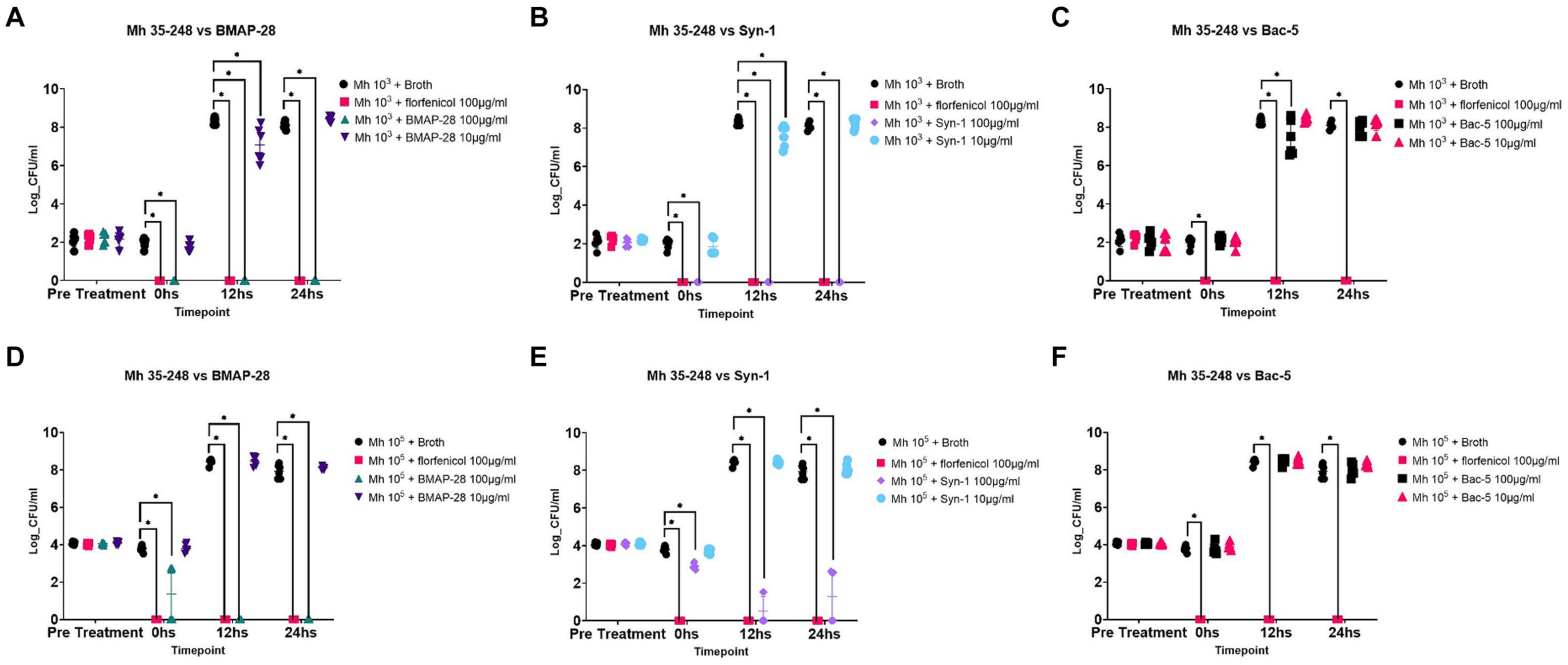
Antimicrobial effect of AMP. *Mannheimia haemolytica* 35-248 was grown to a final concentration of 10^3^ and 10^5^ CFU/mL in Mueller Hinton broth and tested against 100 and 10 μg/mL of each synthetic peptide. Effects of BMAP-28 on Mh **(A,D)**. Effects of Syn-1 on Mh **(B,E)**. Effects of Bac-5 on Mh **(C,F)**. Mh grown in basal media was used as a negative control and Mh tested against 100 μg/mL of florfenicol was used as a positive control. Two trials per experiment were done and the results are shown as the Log CFU/mL of bacteria on each treatment before treatment was applied (pre-treatment) and at times 0, 12, and 24 h. ^*^Significantly different at *p* < 0.05.

Figure 1 shows the log cfu/ml concentration of Mh tested with each treatment at 0, 12, and 24 h. Both BMAP-28 and Syn-1 at 100 μg/mL significantly inhibited Mh at 10^3^ cfu/mL growth at 0, 12, and 24 h when compared to the negative antimicrobial control, whereas both peptides at 10 μg/mL significantly decreased Mh growth at 12 h (Figures 1A,B). Bac-5 at 100 μg/mL had a significant effect on Mh at 12 h, but no effect was seen at the other timepoints. Bac-5 at 10 μg/mL had no effect on bacterial growth (Figure 1C). Florfenicol at 100 μg/mL inhibited bacterial growth, as expected, at all time points. When the AMP were tested against Mh at 10^5^ cfu/mL, BMAP-28 at 100 μg/mL significantly decreased bacterial growth at 0 h, and inhibited growth at 12 and 24 h tested (Figure 1D). Syn-1 at 100 μg/mL significantly decreased bacterial growth at the three time points tested (Figure 1E). Similar to the results of the AMP vs. Mh at 10^3^ cfu/mL, BMAP-28 and Syn-1 at 10 μg/mL, and Bac-5 at either concentration tested, did not inhibit bacterial growth (Figures 1D–F). Florfenicol at 100 μg/mL also showed to be a good positive control for these experimental conditions.

#### MIC and MBC of BMAP-28, Bac-5, and Syn-1 on *Mannheimia haemolytica*

Following the CLSI guidelines, 1 × 10^6^ cfu/ml of each Mh strain was tested against a serial dilution of peptide so that the final concentration ranged between 0.25 and 256 ug/mL, and this was done in a 96-well polypropylene plate. Table 2 shows the AMP MIC and MBC for each bacterial strain. While BMAP-28 and Syn-1 MIC (64 and 128–256 μg/mL) and MBC (64 and 128–256 μg/mL) did not change from strain to strain, the Bac-5 MIC and MBC were lower for the Mh 25-75R5. Overall, the trials were repeatable, and no more than two-fold changes of calculated MIC and MBC were identified.

**Table 2:** AMP MIC and MBC for three *Mannheimia haemolytica* field strains.

Strain	MIC (μg/mL)			MBC (μg/mL)		
	BMAP-28	Bac-5	Syn-1	BMAP-28	Bac-5	Syn-1
Mh 28-32R53	64	128	256	64	256	256
Mh 25-75R5	64	32-16	256	64	64-16	256
Mh 35-248	64	128-64	256-128	64	128	256-128

Using a 96-well plate, 1 × 10^6^ CFU/mL of each Mh strain was tested against a serial dilution of peptide so that the final concentration ranged between 0.2–256 ug/mL. Plates were incubated for 24 h and each treatment MIC was recorded. Wells containing the lowest AMP concentration with visual growth and the next three dilutions were plated for the MBC calculation. AMP, antimicrobial peptide; MIC, minimum inhibitory concentration; MBC, minimum bactericidal concentration; BMAP-28, bovine myeloid antimicrobial peptide 28; Bac-5, bactenecin 5; Mh, *Mannheimia haemolytica*.

#### FICi of the combination of AMPs and florfenicol on *Mannheimia haemolytica* field strains

Using a checkerboard assay, combinations of each AMP and florfenicol were tested against each Mh field strain. Table 3 shows the FICi of each antimicrobial combination and its interpretation for the 3 Mh strains tested. Syn-1 and Bac-5 when combined with florfenicol resulted in an indifference interaction in 1 of 3 and 2 of 3 strains, respectively. Combining BMAP-28 and florfenicol allowed for the use of less antimicrobial to treat all the Mh strains tested, resulting in an addition interaction between both compounds. The same kind of interaction was noted for 2 of 3 and 1 of 3 of the Mh tested when florfenicol was combined with Syn-1 and Bac-5, respectively.

**Table 3:** FICi of AMPs and florfenicol on three *Mannheimia haemolytica* field strains.

Strain	FICi florfenicol +			Interpretation florfenicol +		
	BMAP-28	Bac-5	Syn-1	BMAP-28	Bac-5	Syn-1
Mh 28-32R53	0.53	1	0.625	Addition	Addition	Addition
Mh 25-75R5	0.625–0.75	1–1.25	0.75	Addition	Indifference	Addition
Mh 35-248	0.56	0.75–1.25	1.5	Addition	Indifference	Addition

FICi were calculated using the following formula “FICi = FICa + FICb.” A checkerboard assay was designed with serial dilutions of the AMP and florfenicol, starting from 2 concentrations above the MIC for each antimicrobial. Plates were incubated at 35^+/−^2°C, 5% CO2 for 18–24 h and FICi for each combination of AMP and florfenicol were calculated. FICi, fractional inhibitory concentration index; FICa, fractional inhibitory concentration of compound a; FICb, fractional inhibitory concentration of compound b; MIC, minimum inhibitory concentration; AMP, antimicrobial peptide, BMAP-28, bovine myeloid antimicrobial peptide 28; Bac-5, bactenecin 5; Mh; *Mannheimia haemolytica*.

### Viral assays

#### Effect of BMAP-28, Bac-5, and Syn-1 on BHV-1 and BRSV

**Figure 2:**
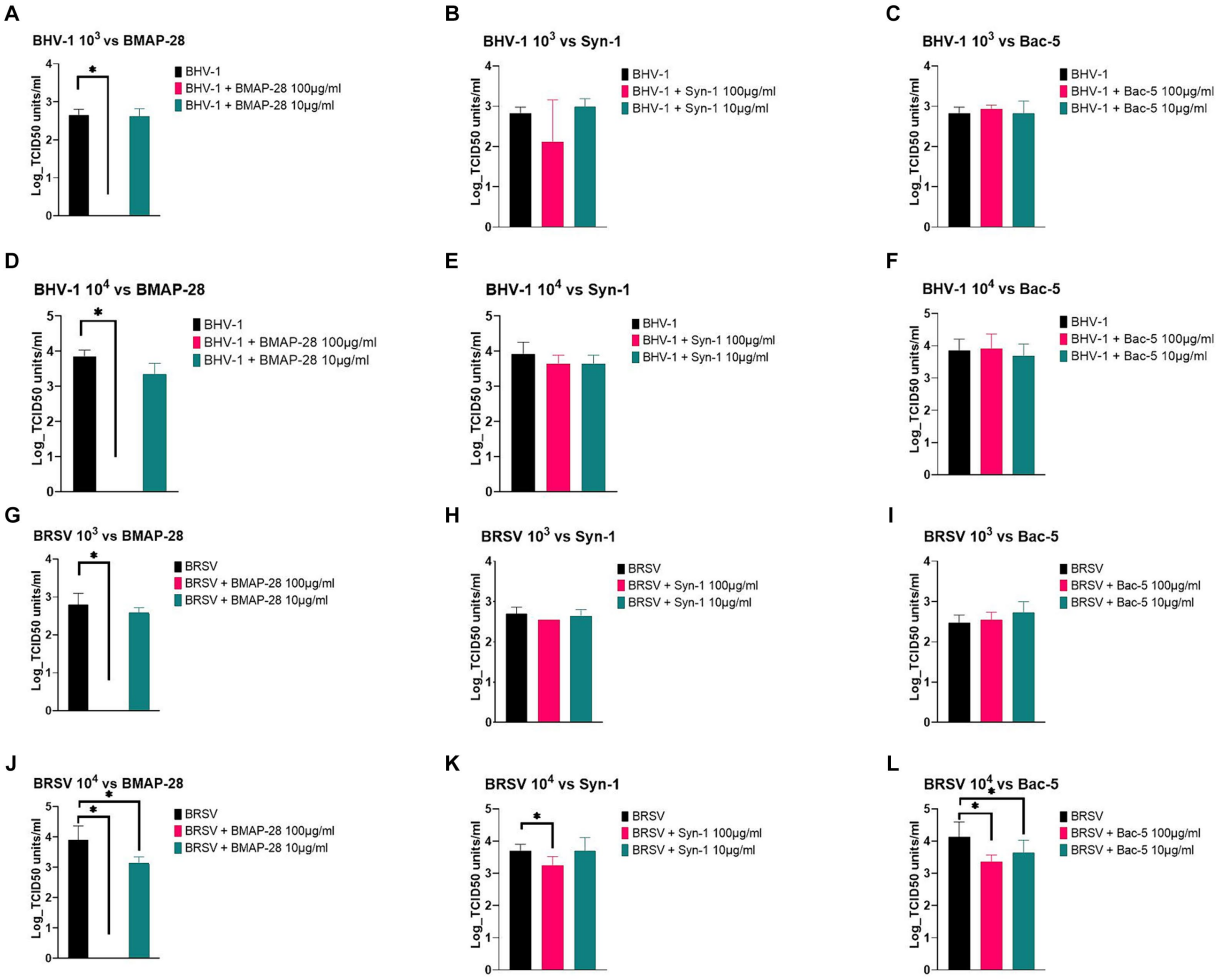
AMP effect on BHV-1 and BRSV replication. BHV-1 and BRSV viruses at 10^3^ or 10^4^ TCID_50_ units/mL were pretreated with a final concentration of 100 or 10 μg/mL of each AMP for 2 h at 37°C and each treatment was used to infect the BK cells previously plated. Plates were incubated for 5 or 7 days, and cytopathic effects were recorded. Effects of BMAP-28 on BHV-1 and BRSV at 10^3^ and 10^4^ TCID_50_ units/ml **(A,D,G,J)**. Effects of Syn-1 on BHV-1 and BRSV at 10^3^ and 10^4^ TCID-50 /mL **(B,E,H,K)**. Effects of Bac-5 on BHV-1 and BRSV at 10^3^ and 10^4^ TCID_50_ units/ml **(C,F,I,L)**. BHV-1 or BRSV grown in basal media was used as a negative control. Two trials per experiment with 3 replicates each were done, and the results are shown as the Log TCID_50_ units/ml of virus on each treatment. ^*^Significantly different at *p* < 0.05.

Viral pathogens were pre-incubated with each cathelicidin for 2 h, to allow the peptides to have an effect on the virus before infecting the cells. Figure 2 summarizes the effects of AMP on BHV-1 and BRSV. At both viral concentrations tested, BMAP-28 at a final concentration of 100 μg/mL was able to completely inhibit BHV-1 replication on BK cells (Figures 2A,D), while the rest of the peptides tested did not have a significant effect on the virus (Figures 2B,C,E,F).

When testing BRSV at 10^3^ TCID_50_ units vs. AMP, BMAP-28 at a final concentration of 100 μg/mL was the only treatment able to completely inhibit viral replication (Figure 2G). The same inhibition was observed for BRSV at 10^4^ TCID_50_ units treated with BMAP-28 at 100 μg/mL (Figure 2J). At the 10^4^ concentration, BMAP-28 at a final concentration of 10 μg/mL as well as Syn-1 at 100 μg/mL and Bac-5 at both concentrations, were able to significantly decrease viral replication on BK cells (Figures 2J–L).

### Cytotoxicity assay

#### Determining AMP cytotoxic effect on mammalian cells

**Figure 3:**
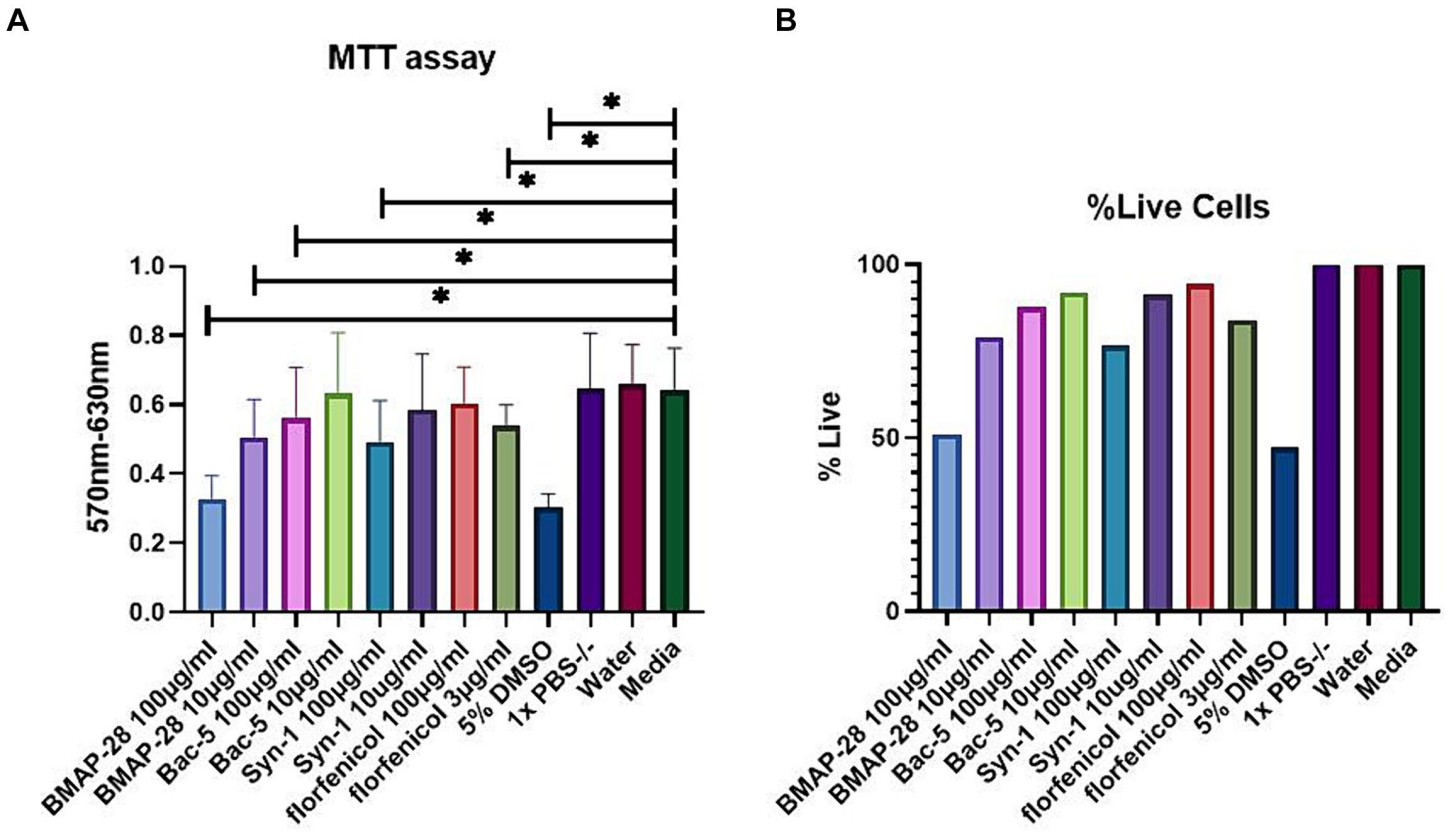
AMP cytotoxicity in BK cells. BK cells were incubated with each treatment for 24 h. MTT solution was then added to each well and the plate was incubated for another 4 h at 37°C. Absorbance was measured using a plate reader at a 570 nm wavelength and background measured at 630 nm was subtracted from signal absorbance. Results are represented as the mean difference of the absorbance read at 570 and 630 nm of each treatment, with 8 replicates per treatment and two trials performed **(A)** or as a percentage of cells that survived AMP treatment relative to the supplemented media treatment **(B)**. DMSO at 5% was used as a positive control and reagent diluents and supplemented media were used as negative controls. ^*^Significantly different at *p* < 0.05.

To determine whether the AMPs used to treat BRD pathogen were toxic to bovine cells *in vitro*, we tested the effect of a final concentration of 100 and 10 μg/mL of each peptide on BK cells. Figure 3A shows the results of the MTT assay. All treatments were compared to the media alone treatment, with BMAP-28 at 100 and 10 μg/mL, Bac-5, and Syn-1 at 100 μg/mL as well as florfenicol at 3 μg/mL showing a significant difference, resulting in an increased toxicity of the treatments when compared to the negative controls. DMSO at 5% also showed a significant difference as expected. Figure 3B shows the percentage of live cells relative to the media treatment which was considered as 100% live cells. This graph shows that the lethal dose 50 of BMAP-28 was 100 μg/mL for BK cells. The percentage of cells that survived treatment with BMAP-28 at 10 μg/mL, Bac-5 at 100 μg/mL, Syn-1 at 10 μg/mL, and florfenicol at 3 μg/mL were 79%, 88%, 76%, and 84%, respectively. This shows that cells can tolerate lower concentrations of BMAP-28 and Syn-1 and that Bac-5 is well tolerated at higher concentrations.

#### Combined effect of BMAP-28 and Bac-5 on *Mannheimia haemolytica*

Table 4 and Supplementary Table S1 show the FICi and MIC of different combinations of BMAP-28 and Bac-5. The FICi of the antimicrobial peptide combinations were < 0.5 for all combinations tested, denoting a synergistic effect (Table 4), and resulting in decreases in the MICs of up to 256-fold for BMAP-28 (0.25 μg/mL) and up to 16-fold for Bac-5 (8 μg/mL; Supplementary Table S1).

**Table 4:** FICi of BMAP-28 and Bac-5 on a MDR *Mannheimia haemolytica* strain.

Strain	FICi	Interpretation
	BMAP-28 + Bac-5	
Mh 35-248	0.19–0.5	Synergism

FICi were calculated using the following formula “FICi = FICa + FICb.” A checkerboard assay was designed with serial dilutions of both AMP ranging from 256 to 0.25 μg/mL. Plates were incubated at 35^+/−^2°C, 5% CO2 for 18–24 h and FICi for each AMP combination were calculated. FICi, fractional inhibitory concentration index; FICa, fractional inhibitory concentration of compound a; FICb, fractional inhibitory concentration of compound b; BMAP-28, bovine myeloid antimicrobial peptide 28; Bac-5, bactenecin 5; MDR, multidrug resistant; AMP, antimicrobial peptide, Mh; *Mannheimia haemolytica*.

## Discussion

AMR is becoming prevalent in bovine respiratory disease (7, 12–15), therefore, there is a critical need to discover new, novel alternatives to antimicrobials. Cathelicidins are AMP that have been explored extensively in a variety of species as alternatives to antimicrobials (21, 23, 25). In cattle, cathelicidins have been used as inflammatory markers for mastitis, tested *in vitro* against mastitis pathogens, and their expression was characterized in endometritis (20, 23, 30, 31), and to our knowledge, we are the second group showing their effect against BRD pathogens (32). The overall purpose of this work was to evaluate the effect of two bovine cathelicidins, BMAP-28, and Bac-5, with different mechanisms of action, and a synthetic version (Syn-1), with established less cytotoxic and enhanced antimicrobial effects (26), on *Mannheimia haemolytica*, BRSV and BHV-1, three known important pathogens in BRD.

In the present study, we tested the effect of these peptides on *Mannheimia haemolytica* growth and showed that BMAP-28 and Syn-1 at 100 μg/mL were able to inhibit the bacteria when tested at 10^3^ cfu/mL. We used 10^3^ cfu/mL inoculum because it corresponds to a 2-log unit difference from the CLSI recommendations for performing antimicrobial susceptibility testing (27). We thought this could be a good starting point for measuring the effect of these peptides. Since we were able to see an effect at this inoculum concentration, we further challenged these peptides’ ability to inhibit bacteria at 10^5^ cfu/mL, an inoculum concentration similar to that of the CLSI recommendations, and saw that BMAP-28 at 100 μg/mL was still able to completely inhibit bacterial growth, while Syn-1 at 100 μg/mL decreased bacterial growth at 10^5^ cfu/mL, but not completely inhibit it. Our results indicate that AMP can be used to inhibit Mh growth. This was also shown by another research group which demonstrated that two synthetically modified bacterial antimicrobial peptides (WRL3 and WK2) and one synthetically modified porcine antimicrobial peptide (PRW4) were able to inhibit Mh growth *in vitro* (32). That study evaluated synthetic molecules which are not naturally occurring, but were designed for antimicrobial and noncytotoxic effects. In contrast, we evaluated naturally occurring bovine peptides, to evaluate them as alternatives to synthetic antimicrobials. Both studies add to the knowledge of the effect of AMP against *Mannheimia haemolytica.*

BMAP-28 and Syn-1 are cationic peptides with an alpha-helical conformation. These kinds of peptides were described to interact with negatively charged bacterial membranes, causing permeabilization and subsequent bacterial death (24, 33). *Mannheimia haemolytica*, a Gram-negative bacterium, has an outer membrane composed of the negatively charged lipopolysaccharide (LPS) which can favor the interaction with the AMP (34). Other researchers have shown, *in vitro*, that bovine myeloid antimicrobial peptides were able to permeabilize *Escherichia coli* and *Salmonella* Typhimurium bacteria, further supporting the effect of these peptides on Gram-negative bacteria (25, 35, 36). Syn-1 was designed by Sahoo et al., and it is a shorter version of BMAP-28. They described that BMAP-28 formed monomers in anionic systems; replacement of leucine residues 3, 6, and 11 with phenylalanine residues in Syn-1 resulted in the formation of dimers which caused Syn-1 to have higher antimicrobial potency against *E. coli*, *Bacillus subtilis,* and *Staphylococcus epidermis* strains when compared to BMAP-28. However, under the conditions of our study, BMAP-28 seems to have a greater effect on Mh than Syn-1, as BMAP-28 at 100 μg/mL was able to completely inhibit growth of 10^5^ cfu/mL of Mh, while Syn-1 decreased growth of 10^5^ cfu/mL of Mh but did not completely inhibit it, showing that the modified peptide is not as potent against Mh.

Although having a positive charge, Bac-5 did not have an effect on Mh, indicating that peptide charge is not the only characteristic needed to exert an effect on Mh. Bactenecins are proline-rich antimicrobial peptides that were shown to translocate to the cytosol of bacteria and inhibit protein synthesis (37, 38). *Mannheimia haemolytica* has the ability to express efflux pumps and proteases which might explain why we did not see an effect of Bac-5 in this study, but more research is warranted to test these hypotheses (39, 40).

To further characterize the antimicrobial effects of these peptides we employed the CLSI guidelines to establish the MIC and MBC of these peptides against 3 Mh field isolates. It is worth noting that the MIC and MBC of Syn-1 were 2-4-fold higher than BMAP-28, further showing that the native peptide performed better than the modified version against this bacterium under these conditions. Antimicrobial susceptibility testing by CLSI guidelines is used to determine a pathogen’s susceptibility to an antimicrobial and help clinicians decide which antimicrobial is best to treat a specific pathogen. We used the same guidelines to describe the AMP antimicrobial effects and decide whether they could be plausibly used as an antimicrobial alternative. Based on our MIC and MBC results, individual peptides used alone against Mh might not be a feasible alternative, as the concentrations needed to inhibit bacteria were toxic to the cells used in this research. Since BMAP-28 outperformed Syn1 and Bac-5, and in an effort to reduce the concentration of the peptide needed to inhibit Mh, we explored the combined effects of BMAP-28 and florfenicol, which is commonly used for BRD treatment. Florfenicol binds the 50s ribosomal subunit of the bacteria, inhibiting protein synthesis and leading to bacterial death, while BMAP-28 permeabilizes the negatively charged membranes of bacterial pathogens (25). Because these two antimicrobials have different mechanisms of action we expected a complementary interaction. The combination of BMAP-28 and florfenicol allowed us to use a lower concentration of each antimicrobial to inhibit bacteria growth, but that reduction was not substantial, with the combination showing an additive but not synergistic interaction.

Viral pathogens are able to cause BRD by themselves and are frequently implicated as the initiators of respiratory disease that is exacerbated by subsequent bacterial infection (41). Therefore, we decided to explore whether these peptides could inhibit BRSV and BHV-1 replication *in vitro*. Under the experimental conditions tested, BMAP-28 outperformed the other peptides, it was able to inhibit viral replication at both viral concentrations tested. BHV-1 and BRSV are both enveloped viruses, with negatively charged membranes and BMAP-28 could have inhibited viral replication by interacting with the membranes and causing permeabilization. Two human cathelicidin analogs, with same alpha-helical structure as BMAP-28, were shown to directly bind to an enterovirus membrane and prevent the virus from entering the cell (42). This could be the mechanism by which BMAP-28 inactivated both viruses tested, but more research is needed to prove that.

Antimicrobial peptides have been shown to be cytotoxic to mammalian cells. The C-terminal hydrophobic tail was shown to be responsible for affinity to mammalian membranes, so researchers have developed truncated version of these peptides in order to mitigate this adverse effect. Our findings were similar to those of others, showing that BMAP-28 has cytotoxic effects on mammalian cells and that modified versions can be less cytotoxic (26). Although the broad antimicrobial effects of BMAP-28 we demonstrated against Mh, BHV-1, and BRSV encourage the concept that cathelicidins can be used as novel antimicrobials, the effective concentration of 100 μg/mL was also the lethal dose (LD_50_) of BMAP-28, indicating that the effective antimicrobial dose of BMAP-28 acting alone might be unacceptably toxic *in vivo*. While BMAP-28 and Syn-1 interact with negatively charged bacterial membranes, Bac-5 targets intracellular processing, inhibiting protein synthesis, which might be the reason the latter peptide showed less cytotoxicity (37, 38). It is worth mentioning that we decided to use a 3 μg/mL concentration of florfenicol as a treatment to compare cytotoxicity levels because that is the antibiotic’s plasma concentration when given either subcutaneously or intramuscularly at the label dose in cattle (43). To our surprise, under the conditions of this study florfenicol at that concentration demonstrated a degree of cytotoxicity that was comparable to BMAP-28 at 10 μg/mL. This suggests that lower concentrations of BMAP-28, which might be effective in combination with other antimicrobials, could have an acceptable toxicity profile *in vivo*.

Because BMAP-28 and Bac-5 have different molecular conformations and mechanisms of action (24, 33, 37, 38), we explored the combinatory effect of both and found out that these peptides work together in a synergistic way, allowing us to reduce the amount of BMAP-28 needed for antimicrobial effect. Panteleev et al. showed that a combination of ChAMP-28 and a mini-ChBac 7.5, two caprine peptides with similar properties as BMAP-28 and Bac-5, had a synergistic effect against various Gram-positive and Gram-negative bacteria (44). These investigators suggested that the synergism could be due to the facilitation of the translocation of one peptide by the other, resulting in an increased antimicrobial effect. BMAP-28’s interaction with the bacterial membrane could facilitate the translocation of Bac-5 inside the cell, enabling its interaction with internal structures or processes.

Bovine cathelicidins could serve as an alternative to conventional antimicrobials in the current period of antimicrobial resistance emergence. Cattle treated for BRD often require more than one round of antibiotics, denoting treatment failure and increasing pressure for antimicrobial resistance development (7). The non-specific mechanisms of actions of these host defense peptides should make them less prone to developing resistance to antimicrobials. Since these cationic peptides interact with negatively charged components of the bacteria (like LPS in Gram-negative bacteria) it seems harder for bacteria to evolve and overcome these effects. In this research, we focused on the antimicrobial effect of cathelicidins against common BRD pathogens, but these peptides have also been shown to exert other immune response effects, like neutrophil recruitment, which can help the host to eliminate an infectious pathogen (18, 19, 45–47). Therefore the antimicrobial effects of cathelicidins *in vivo* will likely be greater than is indicated when they are tested in the absence of host cells in *in vitro* assays. Moreover, knowing that these peptides, with different structures and mechanisms of action, can work together synergistically to kill a bacterial pathogen is a promising finding in this race to find new alternatives to antimicrobials.

The results of this research support the continued investigation of these molecules as novel antimicrobials. The fact that the use of a combination of cathelicidins can lower their effective antimicrobial concentrations to non-cytotoxic levels warrants their evaluation in live animals experimentally challenged by BRD pathogens. Such work would confirm the feasibility of using AMP as an alternative to commercially available antimicrobials and would support the design of new treatments for infectious diseases in livestock.

In conclusion, we investigated the antimicrobial and cytotoxic effects of the bovine cathelicidins BMAP-28, Bac-5, and the BMAP-28 analog Syn-1. BMAP-28 was demonstrated to be the most effective against Mh, BHV-1 and BRSV. Although cytotoxicity was seen at antimicrobial concentrations of BMAP-28, combining this peptide with Bac-5 led to the inhibition of Mh and decreased the concentration of the peptide, which should lower cytotoxicity. These findings make these peptides promising candidates for alternatives to conventional antimicrobials, lowering the risk of antimicrobial resistance emergence.

## Data availability statement

The original contributions presented in the study are included in the article/Supplementary material, further inquiries can be directed to the corresponding author.

## Ethics statement

The animal studies were approved by Mississippi State University Institutional Animal Care and Use Committee. The studies were conducted in accordance with the local legislation and institutional requirements. Written informed consent was obtained from the owners for the participation of their animals in this study.

## Author contributions

SC: Conceptualization, Data curation, Formal analysis, Investigation, Methodology, Validation, Visualization, Writing – original draft, Writing – review & editing. CB: Methodology, Writing – review & editing. MT: Methodology, Writing – review & editing. ML: Methodology, Writing – review & editing. KS: Methodology, Writing – review & editing. AW: Conceptualization, Funding acquisition, Methodology, Resources, Supervision, Writing – review & editing.
