# Supplementary material for: Synthetic antimicrobial peptides Bac-5, BMAP-28, and Syn-1 can inhibit bovine respiratory disease pathogens in vitro
Source: Front Vet Sci. 2024 Aug 12;11:1430919. doi: 10.3389/fvets.2024.1430919 (PMC11345158; doi:10.3389/fvets.2024.1430919)
Supplement: Supplementary file 1 [file Table_1.DOCX]

Supplementary Material

Supplementary Table 1. MIC of BMAP-28 and Bac-5 alone and in combination on a MDR *Mannheimia haemolytica* strain. Using a checkerboard assay, serial dilutions of both AMP ranging from 256 to 0.25 µg/ml were tested against a Mh strain. Plates were incubated at 35+/-2^o^ C, 5% CO_2_ for 18-24 h and MICs for each AMP combination were calculated.

**Strain MIC (µg/ml) MIC in combination (µg/ml) Trial**

**BMAP-28 Bac-5 BMAP-28 Bac-5**

Mh 35-248 64 128 0.25 64 1

2 32 1

4 16 1

16 8 1

0.25 64 2

16 32 2

MIC, minimum inhibitory concentration; BMAP-28, bovine myeloid antimicrobial peptide 28; Bac-5, bactenecin 5; MDR, multidrug resistant; AMP, antimicrobial peptide; Mh, *Mannheimia haemolytica*
